# Neoadjuvant Chemotherapy Followed by Radiofrequency Ablation Prolongs Survival for Ablatable Colorectal Liver Metastasis: A Propensity Score Matching Comparative Study

**DOI:** 10.3389/fonc.2021.758552

**Published:** 2021-10-22

**Authors:** Yizhen Chen, Youyao Xu, Linwei Xu, Fang Han, Yurun Huang, Hang Jiang, Jia Wu, Yuhua Zhang

**Affiliations:** ^1^ Department of Hepatobiliary and Pancreatic Surgery, The Cancer Hospital of the University of Chinese Academy of Sciences (Zhejiang Cancer Hospital), Institute of Basic Medicine and Cancer (IBMC), Chinese Academy of Sciences, Hangzhou, China; ^2^ Zhejiang Chinese Medical University, Hangzhou, China

**Keywords:** colorectal cancer, liver metastasis, neoadjuvant chemotherapy (NAC), radiofrequency ablation, local tumor progression (LTP)

## Abstract

**Background:**

Typically, colorectal liver metastasis (CRLM) is not a candidate for hepatectomy. Radiofrequency ablation (RFA) plays a critical role in unresectable CRLM patients. Nevertheless, high local tumor progression (LTP) and distant metastasis limit the development and further adoption and use of RFA. Neoadjuvant chemotherapy (NAC) has been widely used in resectable CRLM and is recommended by the guidelines. There are no studies on whether NAC can improve the prognosis in ablatable CRLM patients. The present study aimed to determine the feasibility and effectiveness of RFA plus NAC.

**Methods:**

This retrospective cohort included CRLM patients from Zhejiang Cancer Hospital records, who received RFA from January 2009 to June 2020 and were divided into two groups according to the presence or absence of NAC. The Kaplan–Meier method was used to evaluate the 3-year local tumor progression-free survival (LTPFS), progression-free survival (PFS), and overall survival (OS) of the two groups. The propensity score matching was used to reduce bias when assessing survival. Multivariate Cox proportional hazards regression analysis was used to study the independent factors affecting LTPFS, PFS, and OS.

**Results:**

A total of 149 CRLM patients (88 in the RFA alone group and 61 in the plus NAC group) fulfilled the inclusion criteria. Post-RFA complications were 3.4% in the RFA alone group and 16.4% in the plus NAC group. The 3-year LTPFS, PFS, and OS of the RFA only group were 60.9%, 17.7%, and 46.2%, respectively. The 3-year LTPF, PFS, and OS of the plus NAC group were 84.9%, 46.0%, and 73.6%, respectively. In the 29 pairs of propensity score matching cohorts, the 3-year LTPFS, PFS, and OS in the plus NAC group were longer than those in the RFA group (*P* < 0.05). NAC was an independent protective factor for LTPFS, PFS, and OS (*P* < 0.05).

**Conclusions:**

For ablatable CRLM patients, RFA plus NAC obtained a better prognosis than RFA alone. Based on the current results, the application of NAC before RFA may become the standard treatment.

## Introduction

According to recent cancer statistics, colorectal cancer (CRC) is one of the most common cancers and the leading cause of cancer-related deaths worldwide (1, 2). Liver metastases are the most common site of distal spread in colorectal cancer (3). About 50% of CRC patients showed liver metastasis, which severely worsens the overall prognosis of CRC patients (4, 5).

For colorectal liver metastasis (CRLM), the purpose of the treatment is to improve progression-free survival (PFS) and overall survival (OS). Surgical resection is the preferred treatment for CRLM (6). Although the indications for hepatectomy have been expanding, about 80% of CRLM patients are not candidates for hepatectomy (7). Local ablation therapy represented by radiofrequency ablation (RFA) plays a critical role in CRLM patients (8). Although RFA has shown a prognosis similar to hepatectomy in some specific patients (9), the high rate of local tumor progression (LTP) is still an obstacle to using ablation therapy (10).

A significant benefit was obtained in PFS in resectable CRLM patients who underwent surgery after neoadjuvant chemotherapy (NAC) compared with the surgery alone (11). NAC has been routinely used in clinical practice and recommended by guidelines and consensus (12–14). For ablatable CRLM patients, whether NAC followed by RFA can achieve a better prognosis than RFA alone is currently under intensive focus. Therefore, the present study aimed to explore whether NAC can improve the prognosis in ablatable CRLM patients.

## Materials and Methods

### Study Population

We conducted a retrospective review of all cases of ultrasound (US)-guided percutaneous RFA for CRLM in the Cancer Hospital of the University of Chinese Academy of Sciences (Zhejiang Cancer Hospital) from January 2009 to June 2020. All cases were confirmed pathologically as CRLM by liver biopsy during treatment. In a patient with multiple metastases, each metastasis was separately biopsied. The imaging data of these cases (before NAC or RFA) were re-evaluated by a multidisciplinary team composed of surgeons and interventional radiologists.

All initially ablatable CRLM patients undergoing RFA as the primary method of treatment for CLRM were included in this study. Combining the RFA guidelines and expert consensus (15), our center defined percutaneous ablatable criteria as the largest lesion diameter ≤5 cm, and a maximum of five lesions could be ablated simultaneously. The lesion was far away from large blood vessels and major biliary tract, and sufficient liver was remnant after RFA (normal liver ≥ 20%, after chemotherapy ≥ 30%). The primary sites of these cases had been radically resected. All the cases were ablated completely, and the ablation margins were >10 mm. Patients who met any of the following criteria were excluded: 1) the MDT team determined that the patient was initially not suitable for RFA first; 2) extrahepatic metastasis; 3) prior to RFA, other treatments, such as liver resection and hepatic arterial infusion chemotherapy (except NAC), were performed; 4) severe dysfunction of vital organs; and 5) the follow-up time was <6 months.

The eligible patients were divided automatically into two groups for comparison: patients who had not received any treatment before RFA and those who received NAC before RFA. The primary endpoints of this study are LTP-free survival (LTPFS). Secondary endpoints included PFS and OS. This study was approved by the Medical Ethics Committee of the Cancer Hospital of the University of Chinese Academy of Sciences (Zhejiang Cancer Hospital). All patients provided written informed consent. In this study, the first choice for patients in the NAC group was not ablation (but surgical resection or palliative chemotherapy alone). After chemotherapy, the patient and the doctor negotiated to choose RFA for various reasons (for example, the preference of the patient and not suitable for surgery after chemotherapy).

### RFA Procedure

RFA was carried out in collaboration with an interventional radiologist and a hepatobiliary surgeon. The interventional radiologist at our center had >10 years of experience in percutaneous liver ultrasonography and RFA. Percutaneous RFA was performed under general anesthesia. A 16-G bipolar electrode needle was used for RFA. The electrodes were placed in the lesion under US guidance. RFA was performed three times for each lesion site until the rolling endpoint was reached. Consequently, >1 cm ablation margin was achieved. For lesions >3 cm, multiple overlapping ablations were required along with continuous monitoring of local temperature and tissue impedance. During RFA, US in the ablation zone showed high echo, encompassing the lesion area. Contrast-enhanced CT was conducted immediately following the procedure in all cases to assess whether the ablation is complete and no residual viable tumor remained within the ablation zone.

### Definitions

RFA effectiveness is defined as the ablation defect completely surrounding the targeted tumor, and the failure is defined as evidence of residual tumor within 1 cm of the ablation defect. NAC is defined as ablatable CRLM patients who underwent at least one cycle of chemotherapy before RFA. LTP is defined as any new peripheral/nodular enhancement or enlargement of ablation defect within 1 cm of the RFA area (16). LTPFS is defined as the duration interval between the first RFA and the occurrence of LTP. OS is defined as the duration from RFA to the date of death or to the date of the last follow-up. PFS is defined as the duration from RFA to the confirmation of recurrence or death. Objective response was evaluated by CT or MRI scans based on Response Evaluation Criteria in Solid Tumors (RECIST) version 1.1. The modified ablation clinical risk score (CRS) is as follows: node-positive primary tumor, disease-free interval from primary resection to the diagnosis of liver metastasis <12 months, more than one liver tumor, size of largest tumor >3 cm, and carcinoembryonic antigen (CEA) level >30 ng/ml (17). One point was assigned to each item.

### Data Collection and Follow-Up

The baseline characteristics, the course of the disease during RFA (such as complications), NAC regimens, and cycles were obtained from the electronic medical record system. The follow-up was up to May 30, 2021, to obtain the survival status of patients. Each patient was asked to return to the hospital every 3 months after RFA to confirm the progress of the disease by CT or contrast-enhanced ultrasound or MRI. The interval of the follow-up was set at 6 months if the patient was progression-free in the initial 2 years, and the frequency of follow-up was adjusted to once a year if the patients kept the status of progression-free over 5 years.

### Statistical Analysis

All statistical analyses were performed using SPSS statistical software (version 25, SPSS Inc., Chicago, IL, USA). The Pearson’s *χ* ^2^ or Fisher exact test was used to compare the baseline characteristics of the two groups. The LTPFS, OS, and PFS of the two groups were estimated using the Kaplan–Meier method and compared by log-rank test. A Cox proportional hazard multiple regression model was established. First, the univariate analysis was conducted, and related factors (*P* < 0.1) were included in the multivariate analysis. In the multivariate analysis, factors with *P* < 0.05 were considered as independent predictors of LTPPFS, OS, and PFS.

The independent factors related to LTPFS, PFS, and OS and baseline characteristics with significant differences are CEA at diagnosis, timing of metastasis, number of liver metastases, largest diameter, adjuvant chemotherapy after RFA, and modified ablation CRS. The two groups were then formed using a one-to-one nearest neighbor caliper with a width of 0.03.

## Results

### Clinicopathological Characteristics

From 2009 to 2020, a total of 149 CRLM patients met the inclusion criteria: 88 cases comprised the RFA group alone, and 61 cases were in the plus NAC group. The mean age of all patients is 59.1 ± 12.3 years (standard deviation), and the ratio of males to females in the total cohort is 96:53. The clinical characteristics of the two groups of patients are summarized in **Table 1**. Except for age, the timing of liver metastases, number of liver metastases, adjuvant chemotherapy after RFA, and modified ablation CRS, the other baseline characteristics of the two groups did not differ significantly. Nearly half of the patients (49.2%) in the group receiving NAC achieved an objective response. In the plus NAC group, 40/61 patients used the XELOX regimen, 10/61 patients used the FOLFOX regimen, and 11/61 patients used the FOLFIRI regimen. Moreover, 13/61 patients in the plus NAC group were combined with targeted drugs (7 cases of cetuximab and 6 cases of bevacizumab). The median number of cycles of NAC is 4 (3, 6). After propensity score matching, we obtained a one-to-one paired cohort of the plus NAC group and the RFA alone group (29 patients in each group) (**Table 2**). In the matched cohort, there were no longer any significant differences between two groups in any key confounding factors at baseline.

**Table 1:** Baseline characteristics of the patients.

Variables	RFA alone	NAC	*P* ^a^
*N*	88	61	
Age			
≤60/>60	40/48	38/23	0.043
Gender			
Female/male	26/62	27/34	0.065
CEA at diagnosis, ng/ml			
≤30/>30	72/16	44/17	0.161
Location of primary cancer			
Colon/rectum	43/45	26/35	0.453
T stage of primary tumor			
T1–T2/T3–T4	8/80	9/52	0.285
N stage of primary tumor			
N0/N+	24/64	11/50	0.191
Timing of metastasis			
Metachronous/synchronous	78/10	23/38	<0.001
Number of liver metastases			
<3/≥3	82/6	40/21	<0.001
Largest diameter (cm)			
<3/≥3	69/19	41/20	0.126
Adjuvant chemotherapy after RFA			
No/yes	63/25	18/43	<0.001
Response to NAC			
CR + PR/SD + PD		30/31	
Modified ablation CRS^b^			
0–2/3–5	73/15	30/31	<0.001

CR, complete response; PR, partial response; SD, stable disease; PD, progressive disease; CEA, carcinoembryonic antigen.

a The Pearson’s χ^2^ or Fisher exact test was used to compare the basic characteristics of the two groups.

b Node-positive primary tumor + disease-free interval from primary resection to the diagnosis of liver metastasis <12 months + more than one liver tumor + size of largest tumor >3 cm + CEA level >30 ng/ml (mg/L).

**Table 2:** Baseline characteristics of the patients after propensity score matching.

Variables	RFA alone	NAC	*P* ^a^
*N*	29	29	
Age			
≤60/>60	16/13	19/10	0.421
Gender			
Female/male	13/16	12/17	0.791
CEA at diagnosis, ng/ml			
≤30/>30	21/8	22/7	0.764
Location of primary cancer			
Colon/rectum	12/17	10/19	0.588
T stage of primary tumor			
T1–T2/T3–T4	1/28	6/23	0.107
N stage of primary tumor			
N0/N+	8/21	6/23	0.539
Timing of metastasis			
Metachronous/synchronous	22/7	22/7	1.000
Number of liver metastases			
<3/≥3	25/4	26/3	1.000
Largest diameter (cm)			
<3/≥3	18/11	17/12	0.788
Adjuvant chemotherapy after RFA			
No/yes	13/16	13/16	1.000
Modified ablation CRS^b^			
0–2/3–5	22/7	17/12	0.162

CEA, carcinoembryonic antigen.

a The Pearson’s χ^2^ or Fisher exact test was used to compare the basic characteristics of the two groups.

b Node-positive primary tumor + disease-free interval from primary resection to the diagnosis of liver metastasis <12 months + more than one liver tumor + size of largest tumor >3 cm + CEA level >30 ng/ml (mg/L).

### Post-RFA Complications

The analysis of complications after RFA is shown in **Table 3**. The postoperative complication rate of the plus NAC group was higher than that of the RFA alone group (16.4% vs. 3.4%). The postoperative complications of NAC were mainly abdominal infection. Severe complications (CD ≥ 3) occurred in only one case in the plus NAC group, and no severe complications were detected in the RFA alone group. No mortality was observed within 30 days after the RFA.

**Table 3:** Post-RFA complications.

Variables	RFA alone, *N* (%)	NAC, *N* (%)
Overall complications	3 (3.4)	10 (16.4)
Abdominal bleeding	1 (1.1)	0 (0.0)
Abdominal infection	1 (1.1)	7 (11.5)
Liver failure	1 (1.1)	2 (3.3)
Pleural effusion	0 (0.0)	1 (1.7)
Serious complications (CD ≥ 3)	0 (0.0)	1 (1.7)

CD, Clavien–Dindo classification.

### Survival Analysis

**Figure 1:**
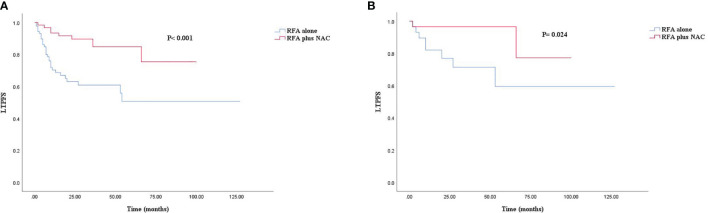
Kaplan–Meier survival curve for LTFS of the radiofrequency ablation (RFA) alone and plus neoadjuvant chemotherapy (NAC) groups. **(A)** Unmatched analyses and **(B)** propensity-score-matched analyses.

**Figure 2:**
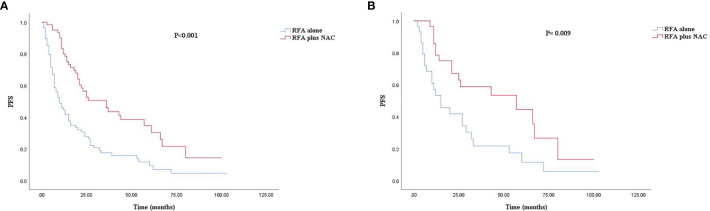
Kaplan–Meier survival curve for PFS of the RFA alone and plus NAC groups. **(A)** Unmatched analyses and **(B)** propensity-score-matched analyses.

**Figure 3:**
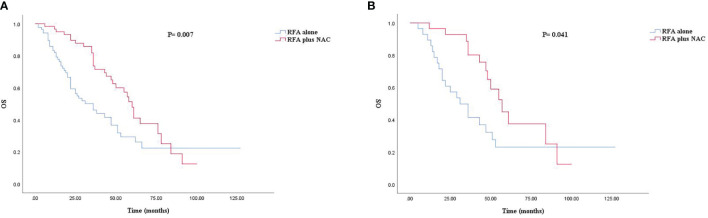
Kaplan–Meier survival curve for OS of the RFA alone and plus NAC groups. **(A)** Unmatched analyses and **(B)** propensity-score-matched analyses.

The median follow-up time for all patients was 30.0 months. As shown in **Figures 1A**, **2A**, and **3A**, the plus NAC group increased the 3-year LTPFS (84.9% vs. 60.9%, *P* < 0.001), PFS (46.0% vs. 17.7%, *P* < 0.001), and OS (73.6% vs. 46.2%, *P* = 0.007) of CRLM patients compared with the RFA alone group. The 3-year LTP rate was 39.1% and 15.1% in the RFA alone and the plus NAC groups, respectively. The results showed that poor LTPFS was independently related to the size of the largest lesion ≥3 cm [hazard ratio (HR), 3.837; 95% CI: 1.973–7.463; *P* < 0.001], and improved LTPFS was independently related to the use of NAC (HR, 0.219; 95% CI: 0.088–0.543; *P* = 0.001) (**Table 4**). The findings revealed that better PFS is independently related to NAC (HR, 0.430; 95% CI: 0.263–0.704; *P* = 0.001) (**Table 5**). Also, enhanced OS is independently related to NAC (HR, 0.427; 95% CI: 0.247, 0.739; *P* = 0.002) and adjuvant chemotherapy after RFA (HR, 0.540; 95% CI: 0.323, 0.902; *P* = 0.019) (**Table 6**). Also, OS is independently related to CEA >30 ng/ml (HR, 1.840; 95% CI: 1.045–3.239; *P* = 0.035). After propensity score matching, the 3-year LTPFS, PFS, and OS of the plus NAC and RFA alone groups were significantly different (96.6% vs. 71.4% in LTPFS, *P* = 0.024, **Figure 1B**; 58.7% vs. 21.7% in PFS, *P* = 0.009, **Figure 2B**; 80.2% vs. 41.5% in OS, *P* = 0.041, **Figure 3B**, respectively).

**Table 4:** Analysis of prognostic factors associated with LTPFS.

Prognostic factor	*n*	Univariate		Multivariate	
		HR (95% CI)	*P*	HR (95% CI)	*P*
NAC					
No	88				
Yes	61	0.280 (0.133–0.592)	0.001	0.219 (0.088–0.543)	0.001
Gender					
Female	53				
Male	96	2.356 (1.119–4.957)	0.024	1.645 (0.765–3.538)	0.203
Age (years)					
≤60	78				
>60	71	1.509 (0.806–2.825)	0.199		
CEA at diagnosis (ng/ml)					
≤30	116				
>30	33	0.749 (0.331–1.693)	0.487		
Primary tumor					
Rectum	80				
Colon	69	1.548 (0.829–2.890)	0.171		
T stage of primary tumor					
T1/T2	17				
T3/T4	132	1.118 (0.398–3.145)	0.832		
LN metastasis					
No	35				
Yes	114	0.601 (0.313–1.151)	0.125		
Timing of metastasis					
Metachronous	101				
Synchronous	48	0.509 (0.242–1.069)	0.075	1.582 (0.669–3.739)	0.296
Number of liver metastases					
<3	122				
≥3	27	1.030 (0.474–2.238)	0.941		
Size of the largest lesion (cm)					
<3	110				
≥3	39	2.677 (1.434–5.000)	0.002	3.837 (1.973–7.463)	<0.001
Adjuvant chemotherapy after RFA					
No	81				
Yes	68	0.323 (0.160–0.652)	0.002	0.486 (0.225–1.050)	0.067
Modified ablation CRS*					
<3	103				
≥3	46	1.201 (0.627–2.301)	0.580		
Post-RFA complications					
No	136				
Yes	13	1.097 (0.390–3.086)	0.861		

HR, hazard ratio; LN, lymph nodes; CEA, carcinoembryonic antigen.

*Node-positive primary tumor + disease-free interval from primary resection to the diagnosis of liver metastasis <12 months + more than one liver tumor + size of largest tumor >3 cm + CEA level >30 ng/ml (mg/L).

**Table 5:** Analysis of prognostic factors associated with PFS.

Prognostic factor	*n*	Univariate		Multivariate	
		HR (95% CI)	*P*	HR (95% CI)	*P*
NAC					
No	88				
Yes	61	0.423 (0.284–0.629)	<0.001	0.430 (0.263–0.704)	0.001
Gender					
Female	53				
Male	96	1.439 (0.963–2.151)	0.076	1.208 (0.798–1.829)	0.372
Age (years)					
≤60	78				
>60	71	1.693 (1.151–2.491)	0.007	1.438 (0.961–2.154)	0.078
CEA at diagnosis (ng/ml)					
≤30	116				
>30	33	1.354 (0.889–2.062)	0.158		
Primary tumor					
Rectum	80				
Colon	69	1.297 (0.888–1.894)	0.178		
T stage of primary tumor					
T1/T2	17				
T3/T4	132	1.705 (0.829–3.507)	0.147		
LN metastasis					
No	35				
Yes	114	1.129 (0.722–1.765)	0.596		
Timing of metastasis					
Metachronous	101				
Synchronous	48	0.684 (0.455–1.030)	0.069	1.379 (0.815–2.332)	0.231
Number of liver metastases					
<3	122				
≥3	27	0.956 (0.588–1.554)	0.855		
Size of largest lesion (cm)					
<3	110				
≥3	39	1.126 (0.732–1.732)	0.588		
Adjuvant chemotherapy after RFA					
No	81				
Yes	68	0.515 (0.350–0.756)	0.001	0.721 (0.453–1.146)	0.167
Modified ablation CRS*					
<3	103				
≥3	46	0.977 (0.655–1.457)	0.908		
Post-RFA complications					
No	136				
Yes	13	0.925 (0.467–1.834)	0.824		

HR, hazard ratio; LN, lymph nodes; CEA, carcinoembryonic antigen.*Node-positive primary tumor + disease-free interval from primary resection to the diagnosis of liver metastasis <12 months + more than one liver tumor + size of largest tumor >3 cm + CEA level >30 ng/ml (mg/L).

**Table 6:** Analysis of prognostic factors associated with OS.

Prognostic factor	*n*	Univariate		Multivariate	
		HR (95% CI)	*P*	HR (95% CI)	*P*
NAC					
No	88				
Yes	61	0.541 (0.343–0.855)	0.009	0.427 (0.247–0.739)	0.002
Gender					
Female	53				
Male	96	1.422 (0.887–2.278)	0.144		
Age (years)					
≤60	78				
>60	71	1.747 (1.106–2.758)	0.017	1.551 (0.950–2.533)	0.079
CEA at diagnosis (ng/ml)					
≤30	116				
>30	33	1.602 (0.996–2.575)	0.052	1.840 (1.045–3.239)	0.035
Primary tumor					
Rectum	80				
Colon	69	1.288 (0.824–2.011)	0.267		
T stage of primary tumor					
T1/T2	17				
T3/T4	132	1.325 (0.610–2.881)	0.477		
LN metastasis					
No	35				
Yes	114	1.657 (0.929–2.956)	0.087		
Timing of metastasis					
Metachronous	101				
Synchronous	48	0.777 (0.483–1.250)	0.299		
Number of liver metastases					
<3	122				
≥3	27	0.901 (0.504–1.611)	0.726		
Size of largest lesion (cm)					
<3	110				
≥3	39	1.420 (0.868–2.323)	0.162		
Adjuvant chemotherapy after RFA					
No	81				
Yes	68	0.489 (0.310–0.771)	0.002	0.540 (0.323–0.902)	0.019
Modified ablation CRS					
<3	103				
≥3	46	1.506 (0.954–2.377)	0.079	1.772 (0.990–3.171)	0.054
Post-RFA complications					
No	136				
Yes	13	0.853 (0.392–1.856)	0.688		

HR, hazard ratio; LN, lymph nodes; CEA, carcinoembryonic antigen.

## Discussion

Liver metastasis worsens the prognosis of CRC patients. As a commonly used minimally invasive approach, RFA is characterized by less trauma, fewer complications, and a short hospital stay, which has been widely used in the local control of CRLM (18). For unresectable CRLM patients, RFA with postoperative chemotherapy achieved a better prognosis than chemotherapy alone (19, 20). However, compared with surgical resection, RFA for CRLM was considered to have a high recurrent rate, irrespective of being extrahepatic or intrahepatic, and to have a low survival rate (21). Therefore, for patients who need local treatment, prolonging their survival is crucial. Most of the current consensus definitions are as follows: I) unresectable liver lesions; II) combination with hepatectomy; III) patients with significant medical comorbidities or World Health Organization (WHO) poor performance status (performance status > 1); IV) a small (<3 cm) solitary lesion, which would otherwise necessitate a major liver resection; and V) patient preference (8, 22). NAC has been widely used in the clinical treatment of CRLM and is recommended by guidelines and consensus (12–14). The multivariate analysis of retrospective studies suggested that NAC is an independent predictor of survival in resectable CRLM patients, which increases the probability of R0 and the remaining liver volume after surgical resection (23). In addition, NAC improves the prognosis of CRLM patients and prolongs survival (24). The guidelines and consensus were focused on NAC for resectable CRLM patients. However, no studies were associated to ablatable CRLM. The current study focused on investigating whether NAC followed by RFA could achieve a better prognosis than RFA alone for ablatable CRLM patients.

The 3-year LTPFS, PFS, and OS of the RFA only group were 60.9%, 17.7%, and 46.2%, respectively. Surprisingly, the two groups have significant differences in LTPFS, PFS, and OS, and the plus NAC group showed an improved prognostic advantage (84.9%, 46.0%, and 73.6%, respectively). NAC was associated with LTPFS, PFS, and OS, which reduced the risk of LTP by 88% (HR = 0.219, *P* = 0.001), the risk of systemic disease progression by 53% (HR = 0.430, *P* = 0.001), and the risk of death by 53% (HR = 0.427, *P* = 0.002). The credibility of these research results was confirmed again by propensity score matching analysis. The analysis simulates the randomization of prospective studies and reduces the bias caused by confounding variables. To the best of our knowledge, this is the first comparative retrospective study to explore the prognostic advantage of RFA plus NAC. Previous single-arm studies were only for patients, who were initially unsuitable for local ablation treatment and were finally treated with RFA after conversion therapy (25, 26).

Nearly 50% of the basic clinical characteristics were significantly different between the two groups in this study, which might have an impact on the prognosis. Age >60 years, synchronous metastases, number of liver metastases >2, and modified ablation CRS >2 are considered as factors of poor prognosis for CRLM, while adjuvant chemotherapy after RFA is considered as a factor for protecting prognosis (13, 17). The RFA only group consisted of a high proportion of people >60 years old or who did not receive adjuvant chemotherapy after RFA. The plus NAC group also consisted of a high proportion of synchronous metastases, a number of liver metastases >2, and modified ablation CRS >2. Interestingly, after multivariate analysis of LTPFS, PFS, and OS, except for adjuvant chemotherapy after RFA, other bias factors did not have a critical impact on the prognosis. The effect of adjuvant chemotherapy after RFA on the prognosis could not be resolved in the retrospective study, and further prospective trials are essential.

The high LTP rate is an obstacle to the widespread use of RFA (27). The 3-year LTP rate of the RFA alone group was nearly three times higher than that in the plus NAC group. In the multivariate analysis of LTPFS, the use of NAC and the size of the lesion have a significant impact on LTPFS. Previous studies have confirmed that the size of the lesion affects the incidence of LTP after RFA in CRLM patients (28). The efficacy of RFA will reduce with the increase in lesion size (27, 29). The proportion of the largest diameter >3 cm in the plus NAC group was higher, while the LTPFS was better than the RFA alone group. In this study, nearly half of the CRLM patients in the plus NAC group achieved an objective response, i.e., reduced lesion size, and associated improvement in LTPFS.

Only NAC was the single variable of significance in multivariate analysis of PFS. The improved PFS due to NAC reduced the lesion diameter and eliminated the micrometastatic sites, which ultimately reduced the local and distant recurrence rates. For resectable CRLM patients, surgical resection plus NAC could also improve PFS (11, 30).

In the multivariate analysis of OS, NAC, CEA, and adjuvant chemotherapy have a significant impact on OS. In CRLM patients undergoing RFA, poor survival prognosis is associated with high LTP (31). Also, as expected, NAC improves the prognosis by reducing local recurrence and distant metastasis. This result is more attractive than NAC in resectable CRLM. The high CEA is associated with poor OS. In 2016, Shady et al. proposed a modified ablation CRS score suitable for predicting the prognosis of OS and LTP for RFA, which was applied in the present study (17). Both the modified ablation CRS and the classic CRS assign CEA as one of items (32). Although postoperative adjuvant chemotherapy did not improve PFS and LTPFS, it improved the OS. CRLM patients require adjuvant chemotherapy after surgical resection, which is also recommended by the guidelines (13, 14). Herein, we proposed that adjuvant chemotherapy should also be routinely carried out after RFA, which could further reduce the risk of recurrence, necessitating prospective trials.

The postoperative complications in the plus NAC group were higher in this study. The use of NAC, followed by surgical resection, had higher complications than the surgery alone group (33). However, several studies demonstrated that these complications did not affect the prognosis of patients (34). A retrospective study included CRLM patients who underwent surgical resection from 1996 to 2006 and were divided into the NAC group and surgery alone group. The univariate and multivariate analyses showed that there was no difference between the two groups in terms of morbidity (*P* = 0.81), mortality (*P* = 0.29), PFS (*P* = 0.25), and OS (*P* = 0.30) (35). This phenomenon is consistent with the multivariate results of this study and the complications caused by NAC that will not affect the prognosis of patients.

The present study has some limitations. First, this is a retrospective single-center study. Differences were detected in the clinical baseline characteristics of the two groups. Although propensity score matching and multivariate analysis have resolved the possible deviations caused by clinical baseline imbalance, the reduced sample size after matching may affect the results. Second, the sample size of this study is small, which might affect the results. Third, there may have been patients who were technically “ablatable” and were given chemotherapy by their oncologist. However, follow-up imaging may have demonstrated progression or complete remission which ultimately resulted in no RFA. These patients cannot be included in the study due to limitations of retrospective study. Finally, RAS mutation status is an important prognostic tool for the determination of LTPFS and OS. Also, limited to the flaws of retrospective study, the authors do not include RAS mutation status in this study.

The results of this study are positive, which is hopeful for patients requiring local ablation. In the future, there are several aspects that need to be further elucidated. For example, 1) the interval between NAC and RFA should be set within a few weeks to ensure safety but not affect the treatment and 2) the optimal cycle of NAC and whether there are advantages to adding targeted drugs. In summary, NAC reduces LTP and prolongs PFS and OS in CRLM patients. Although NAC increases postoperative complications, it does not affect the long-term prognosis. The application of NAC before RFA deserves further evaluation as it is speculative based on the results of this study to imply that NAC before RFA will be the standard of care. However, the application of NAC before RFA needs further verification by prospective clinical trials. Therefore, large-sample prospective, double-blind controlled trials are required to substantiate the feasibility of NAC, followed by RFA.

## Data Availability Statement

The raw data supporting the conclusions of this article will be made available by the authors, without undue reservation.

## Ethics Statement

The studies involving human participants were reviewed and approved by the Medical Ethics Committee of the Cancer Hospital of the University of Chinese Academy of Sciences (Zhejiang Cancer Hospital). The patients/participants provided their written informed consent to participate in this study.

## Author Contributions

All authors contributed to the study conception and design. Material preparation and data collection were performed by YC, JW, and LX. Data analysis was performed by YC, YH, HJ, YX, and FH. The first draft of the manuscript was written by YC. The review and editing of the article were performed by YZ and JW. All authors commented on previous versions of the manuscript. All authors contributed to the article and approved the submitted version.

## Conflict of Interest

The authors declare that the research was conducted in the absence of any commercial or financial relationships that could be construed as a potential conflict of interest.

## Publisher’s Note

All claims expressed in this article are solely those of the authors and do not necessarily represent those of their affiliated organizations, or those of the publisher, the editors and the reviewers. Any product that may be evaluated in this article, or claim that may be made by its manufacturer, is not guaranteed or endorsed by the publisher.
